# Socioeconomic inequalities in the use of medical consultation services in Peru, 2019

**DOI:** 10.1186/s12939-024-02099-2

**Published:** 2024-01-20

**Authors:** Renato Díaz-Ruiz, Rodrigo Vargas-Fernández, Carlos Rojas-Roque, Akram Hernández-Vásquez

**Affiliations:** 1 Universidad Científica del Sur, Lima, Peru; 2 University of York, York, UK; 3 Centro de Excelencia en Investigaciones Económicas y Sociales en Salud, Vicerrectorado de Investigación, Universidad San Ignacio de Loyola, Lima, Peru

**Keywords:** Socioeconomic disparities in Health, Ambulatory care, Health services, Peru

## Abstract

**Background:**

Socioeconomic inequalities in the population influence access to health services and constitute a challenge for health systems, especially in low- and middle-income countries. In Peru, an increase in the use of medical services has been estimated; however, the study of inequalities in the use of medical services is limited. Therefore, the objective of this research was to analyze and decompose socioeconomic inequalities in the use of medical consultation services in Peru.

**Methods:**

A cross-sectional analytical study was conducted using data from the National Household Survey 2019. The outcome variable was the use of a consultation attended by a physician in the last 4 weeks in persons who presented symptom or discomfort, illness, relapse of chronic disease and/or accident. Concentration curves and Erreygers concentration indices were used to determine socioeconomic inequalities, and a generalized linear regression model was used for the decomposition analysis of inequalities.

**Results:**

A total of 52,715 persons were included in the study. The frequency of medical consultation was 25.4% (95% confidence interval: 24.8 − 26.1%). In the inequality analysis, it was found that the use of medical consultations was concentrated among the wealthiest individuals. The main contributing factors were having another type of health insurance (social health insurance [EsSalud], private health insurance, health provider, the Armed Forces, and the Police), residing in an urban area, belonging to the richest wealth quintile, having a chronic disease, and residing in the highlands of Peru.

**Conclusions:**

Based on our findings, government institutions seeking to achieve equitable access to health services should consider the main factors contributing to this inequality in the formulation of strategies to lessen the negative impact of inadequate disease control in the population.

## Introduction

Inequality in access to health care is the difference, variation or disparity that exists between individuals or population groups in accessing health services [1, 2]. In Peru, despite the fact that health is a universal right and is considered by the Political Constitution within the social and economic rights [3], there is inequality in access to health care [4]. In 2009, the Framework Law on Universal Health Insurance was approved, which contemplates the principle of equity, whereby the State provides greater health care coverage to the most vulnerable and those with the least economic resources [5]. Thus, Peru is progressively seeking to ensure that everyone has health insurance that allows them to access and finance preventive, promotional, recuperative and rehabilitation services if required [5].

The use of health services by individuals, such as medical consultation, is one of the most common ways to assess health inequalities [4, 6]. Some studies that have used Andersen’s theoretical model [7, 8] to analyze such inequalities have reported an association between predisposing characteristics (e.g., sex, age, marital status), enabling resources (e.g., health coverage, employment, income), and health care use [9]. For example, in Iran and China, people with higher socioeconomic status have been reported to have used outpatient medical services more frequently [10, 11], whereas in Sweden, higher rates of utilization of general practitioner and specialist physician consultations by people with higher socioeconomic status have been reported [6]. A relevant determinant of this inequality is the coverage of complementary health insurance, which would explain the inequality up to 74.05% in rural areas and 147.63% in urban areas [10]. Income is another determinant that contributes up to 124.6% to inequality in the use of outpatient services [11].

Similarly, evidence from studies conducted in Latin America and the Caribbean (LAC) reports that people with greater economic resources have higher use of health services [12–15]. In LAC, where health systems are fragmented and segmented [16], private health insurance is an important factor in explaining inequality in favor of people with higher socioeconomic status [12–15]. In Peru, a similar pattern was reported in which people with higher socioeconomic status have higher utilization of medical consultations associated with illnesses or accidents, with household spending and health insurance being the factors that contribute most to inequality [4]. In 2015, the Pan American Health Organization (PAHO) reported that 32.7% of people in the highest quartile of per capita expenditure used medical consultations in the last month prior to the survey, while the percentage is 24.6% for people in the lowest quartile [17].

In Peru, the National Household Survey on Living Conditions and Poverty (ENAHO – acronym in Spanish) is one of the main surveys that allows studies on the social determinants of health [18]. According to PAHO, the ENAHO reported an increase in the use of medical consultations of 10.7 percentage points between 2010 and 2015 [17]. Despite this, there is little information on socioeconomic inequalities in the utilization of the medical consultation service. This study aims to analyze and decompose socioeconomic inequalities in medical consultation service utilization in Peru using the ENAHO 2019. The decomposition identifies the factors that contribute most to inequality. The results can help decision makers to design measures aimed at universal access to health services.

## Methods

### Study setting

Peru is home to 33 million people, of which almost 30% live in Lima, the capital city. The rest of the population lives in coastal regions, highlands and jungle [19, 20]. The Peruvian health system is fragmented and segmented into four sectors. First, Seguro Integral de Salud (SIS) aims to provide coverage to people living in poverty. The SIS is financed by public taxes and covers almost 65% of the total population [21]. Next, the Social Health Insurance (EsSalud) provides health and pension coverage to workers [21]. EsSalud is financed through payroll deductions and depends on the Ministry of Labor. In addition, the Ministry of Defense finances insurance for members of the Armed Forces and the Police [21]. Finally, there are several private insurers covering almost 10% of the population, with some overlap of coverage with EsSalud [21]. Hospital centers and health centers are concentrated in urban areas; there are also differences in the accessibility and availability of health services according to regions in Peru. Therefore, timely access to health care is limited for those living in rural settings [22, 23].

In addition, the World Health Organization (WHO) has developed the Human resources density ratio in health, which was 44.5 physicians, nurses and midwives per 10,000 inhabitants to provide essential health services to the population [24]. However, the density of human resources in Peru according to the year 2021 is 16.79 physicians, 20.30 nurses and 6.36 obstetricians per 10,000 inhabitants. Furthermore, the highest density of physicians per 10,000 inhabitants in Peru is found in the Coast (Callao, Moquegua and Lima) and the Highlands and Jungle contain the departments with the lowest density of physicians per 10,000 inhabitants [25].

### Study design and data source

This study used secondary data from the ENAHO 2019, prepared by the National Institute of Statistics and Informatics (INEI – acronym in Spanish). The ENAHO is an annual survey representative of the Peruvian population that collects information on housing and household characteristics, as well as characteristics of household members on education, health, employment and economic income, household expenditures, among others [26]. Data were used from the housing and household characteristics module (base enaho01-2019-100), the household member characteristics module (base enaho01-2019-200), the education module (base enaho01a-2019-300), the health module (base enaho01a-2019-400), and the summary module (base sumaria-2019-2019). Documents related to the ENAHO 2019 design, instruments, procedures, and manuals are published on the INEI website https://proyectos.inei.gob.pe/microdatos/.

### Study population and sample

The ENAHO 2019 used a probabilistic, area-based, stratified, multistage and independent sample in each study department. Data collection was conducted through a survey of all usual residents of the household with direct method by previously trained enumerators. The informant could be the head of household, spouse, percipients, and persons 18 years of age or older [27]. The sample size of the ENAHO 2019 was 36,994 dwellings (23,346 dwellings in urban areas and 13,648 in rural areas) and the data are freely available on the INEI website.

**Fig. 1:**
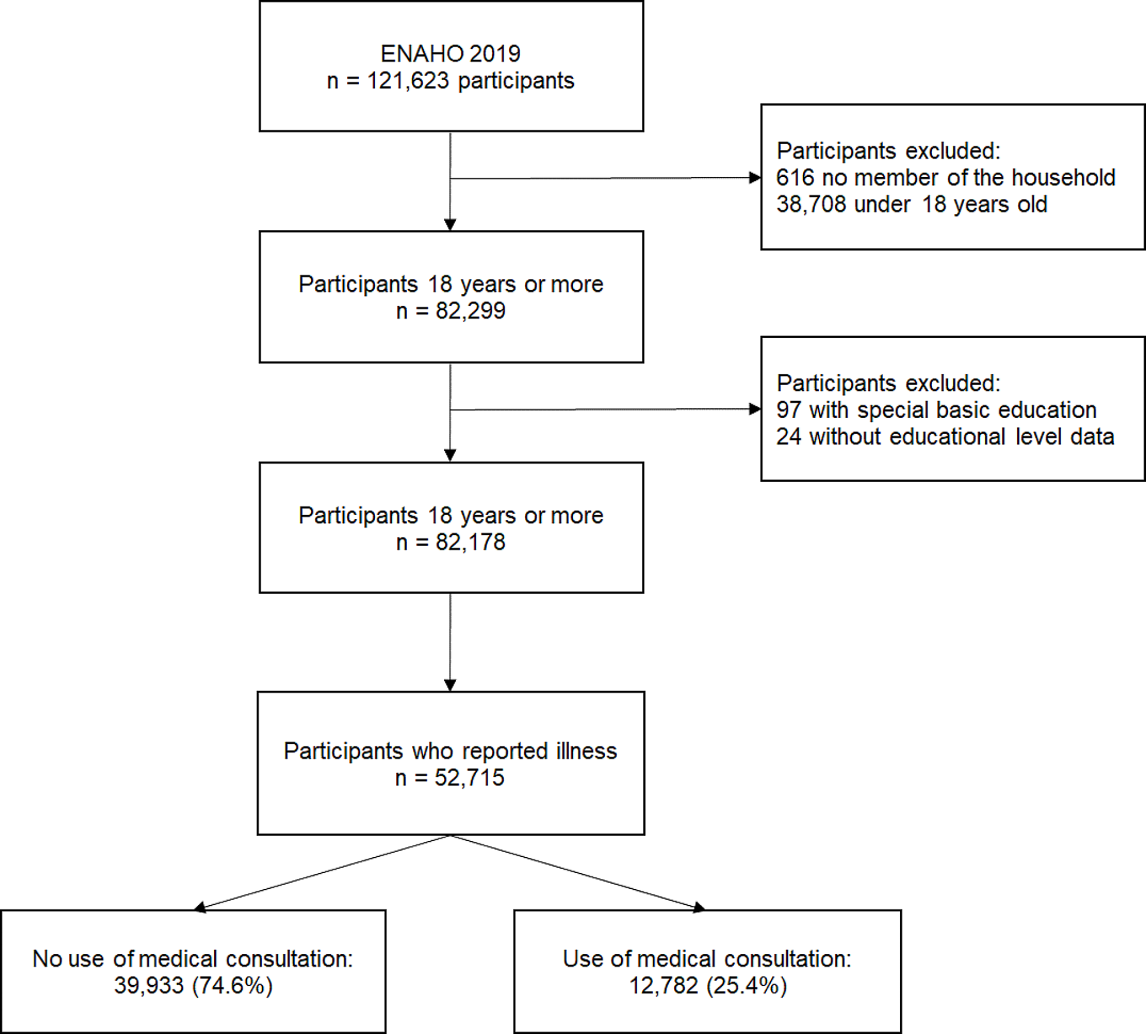
Flowchart of the selection of adults included in the study

This study considered a final subsample of 52,715 persons who reported illness by answering affirmatively to any of the following questions of the ENAHO 2019 health module: “In the last 4 weeks, did you present any (a): symptom or discomfort (cough, headache, fever, nausea); (b) illness (flu, colitis, etc.); (c) relapse of chronic illness; (d) accident?”. Household members aged 18 years or older were included and those with incomplete data on the variables of interest or who reported having special basic education were excluded. Details of the selection of persons for the subsample are presented in Fig. 1.

### Variables

#### Outcome variable

The dependent variable is the use of medical consultation in the last 4 weeks in persons who presented any symptom or discomfort, illness, relapse of chronic disease and/or accident. The dependent variable was dichotomized as 1 in case of use of medical consultation and 0 otherwise.

#### Independent variable

The independent variable is the household’s monthly per capita expenditure, measured in soles. Monthly per capita expenditure is a proxy variable for ordering households according to their level of wealth. Since household expenditures are more stable compared to household income, the structure of expenditures and consumption expenditures can reflect household economic wealth [28]. In addition, INEI and other national institutions jointly estimated poverty levels using household expenditures [29]. Monthly per capita expenditure was constructed by dividing the total annual household expenditure by twelve months and the total number of household members.

For the descriptive analysis, expenditure was expressed as quintiles (I: poorest, II: poorer, III: middle, IV: richer, V: richest). For the analysis of concentration indexes and concentration curves, expenditure was expressed as a continuous variable through monthly per capita expenditure. For the decomposition analysis, expenditure was expressed as monthly per capita expenditure and as quintiles.

### Covariates

Covariates were selected based on previous studies [4, 6, 10–15] and based on Andersen’s theoretical model [7, 8]. The theoretical model indicates that the use of health services is a function of three main factors: predisposing factors, enabling factors, and need factors. Predisposing factors are composed of sociodemographic characteristics, social structure characteristics, and health beliefs of individuals. Enabling factors correspond to people’s means and capacity to use health services at both the individual and community levels. The need factors correspond to variables that predispose to the use of health services due to the individual’s health problems.

The predisposing characteristics considered in the analysis were age according to the classification of the Target Groups for the Comprehensive Care Programs of the Peruvian Ministry of Health (18 to 29 years / 30 to 59 years / 60 years and older) [30]; sex (male / female); language or mother tongue learned in childhood (non-native / native); educational level (up to primary / secondary / higher). The enabling resources selected in the study are geographic region (coast / highlands / jungle); area of residence (rural / urban) and health insurance (no insurance / Seguro Integral de Salud / others). Others category was made up of EsSalud, private health insurance, health provider entity, Armed Forces and the Police. The need factors included in the analysis are suffering from a chronic disease or discomfort such as arthritis, hypertension, asthma, rheumatism, diabetes, tuberculosis, human immunodeficiency virus (HIV), cholesterol, etc. (yes / no); presence of any permanent limitation or difficulty (yes / no) that prevents or hinders you from carrying out your daily activities normally, such as limitation to move or walk, to use arms or legs; limitation to see, even when wearing glasses; limitation to speak or communicate, even when using sign language or other; limitation in hearing, even when using hearing aids; limitation in understanding or learning (concentrating and remembering); and limitation in relating to others, by their thoughts, feelings, emotions or behaviors, according to the questions of the Washington Group on Disability Statistics [31].

### Statistical analysis

Through Stata® v17.0 software (Stata Corporation, College Station, Texas, USA) the statistical processing and analysis was performed, specifying the sampling characteristics of the ENAHO 2019 including the sampling design and the expansion factor. A *p* < 0.05 was considered statistically significant in all tests. After downloading the 2019 “ENAHO Metodología ACTUALIZADA” bases (https://proyectos.inei.gob.pe/microdatos/) that included our study variables, we generated a new database that joined the downloaded databases to perform data management and statistical analysis. Nominal variables were described using absolute frequencies, relative frequencies and their 95% confidence interval (CI).

Inequalities in the use of the medical consultation service were analyzed using the concentration curves (CC) and Erreygers concentration indexes (ECI) described by Wagstaff et al. [32]. The CCs describe the relationship between the cumulative percentage of people studied according to the wealth index and the cumulative percentage of medical consultation service use in relation to the diagonal line of equality. Inequality is expressed according to the concavity of the curve and reflects greater inequality the further the CC is from the equality line. If it is below or above the equality line, there is greater use of medical consultation by people with a higher or lower wealth index, respectively. The ECI is a coefficient whose values range between − 1 and 1, with zero being the absence of inequality between groups, the positive value concentrates the distribution in the richest and a negative value in the poorest [33].

The decomposition analysis was performed using Van Doorslaer’s methodology [34] by means of a generalized linear regression model, based on a linear approximation of the partial effects of each factor evaluated on the sample means. This measure estimates the elasticity, concentration index, contribution and percentage contribution for each independent variable to be included in the analysis.

To decompose the inequalities, the elasticity, the concentration index, the absolute and relative contribution of the concentration index to inequality were estimated for each independent variable. Elasticity denotes the change in the outcome of interest associated with a one-unit change in the independent variable [34]. A positive and negative sign on the elasticity indicates an increasing or decreasing change in the outcome of interest in association with a positive change in the independent variable [34]. The concentration index represents the concentration ratio of the independent variables with reference to the wealth index. A positive/negative value means that the variable of interest is more prevalent among richer/poorer individuals [34]. Finally, the absolute and relative contribution represent the absolute and relative contribution of each independent variable, included in the model, to the overall socioeconomic inequality in the variable of interest. A positive/negative contribution or percentage contribution in a variable result in an increase/decrease in observed socioeconomic inequality [34].

### Ethical considerations

The Ethics and Research Committee of the Universidad Científica del Sur approved the project of the present study (registration code 680-2021-POS50). However, the study used a secondary database that is freely available on the INEI’s microdata web page (https://proyectos.inei.gob.pe/microdatos/). In the databases, respondents are coded so as not to be identified.

## Results

A total of 52,715 persons who reported a health problem during the last four weeks were included in the study. Table 1 describes the demographic and socioeconomic characteristics of the study population. More than half of the population (51.9%) was between 30 and 59 years of age, and the majority of the population was female (56.3%). Approximately a quarter of the population (23.6%) spoke a local or native language (e.g., Quechua or Aymara), while a third of the population had attained primary education (34.3%) and 36.1% had attained secondary education. Almost six out of ten people have at least one chronic disease (59.7%), while the vast majority of the population reported no physical limitations (92.3%). The majority of the population resides in urban areas (77.5%) and approximately a quarter of the population has no health coverage (24.7%).

**Table 1:** Characteristics of the participants included in the study (*n* = 52,715)

Characteristics	n		% (95% CI)
Age group (in years)			
18–29	10,402		21.4 (20.8–21.9)
30–59	27,490		51.9 (51.3–52.5)
60 or older	14,823		26.7 (26.1–27.3)
Sex			
Male	23,182		43.7 (43.2–44.2)
Female	29,533		56.3 (55.8–56.8)
Mother tongue			
Non-native	38,703		76.4 (75.4–77.4)
Native	14,012		23.6 (22.6–24.6)
Educational level			
Up to primary	21,051		34.3 (33.6–35.0)
Secondary	17,482		36.1 (35.3–36.8)
Higher	14,182		29.7 (28.9–30.5)
Chronic disease or discomfort			
No	21,381		40.3 (39.6–41.0)
Yes	31,334		59.7 (59.0-60.4)
Presence of any permanent limitation or difficulty			
No	48,347		92.3 (91.9–92.7)
Yes	4368		7.7 (7.3–8.1)
Geographic region			
Coast	21,988		54.3 (52.7–55.8)
Highlands	20,853		34.8 (33.3–36.4)
Jungle	9874		10.9 (10.1–11.8)
Area of residence			
Rural	19,680		22.5 (21.8–23.2)
Urban	33,035		77.5 (76.8–78.2)
Health insurance			
No	11,820		24.7 (24.0-25.4)
Seguro Integral de Salud	27,040		46.1 (45.2–47.0)
Others*	13,855		29.2 (28.4–30.0)
Wealth quintile			
I (Poorest)	13,682		20.4 (19.7–21.2)
II	11,309		20.6 (19.8–21.4)
III	10,534		21.5 (20.7–22.4)
IV	9629		20.6 (19.8–21.4)
V (Richest)	7561		16.8 (16.0-17.7)

CI: confidence Interval

*Others category was made up of EsSalud, private health insurance, health provider entity, Armed Forces and the Police

Estimates include the expansion factor and sample design characteristics of the ENAHO 2019

Table 2 reports the frequency of medical consultation among study participants who reported a health problem during the previous four weeks. Overall, the frequency of medical consultation was 25.4% (95% CI: 24.8 − 26.1%). The frequency of medical consultation was different according to age, sex and language (*p* < 0.001). Women reported attending more medical consultations compared to men (27.9% vs. 22.2% respectively, *p* < 0.001). More medical consultations were reported in non-native speakers than in native speakers (26.6% vs. 21.6%; respectively, *p* < 0.001). In individuals with higher education, those living on the coast, those living in the urban area and the wealthier, a higher frequency of medical consultation was evidenced (*p* < 0.001).

**Table 2:** Frequency of use of medical consultation in Peru, ENAHO 2019

		Use of medical consultation				
Characteristics		No (*n* = 39,933)		Yes (*n* = 12,782)		
		% (95% CI)		% (95% CI)		*p* value*
Overall		74.6 (73.9 − 75.2)		25.4 (24.8 − 26.1)		
Age group (in years)						
18–29		81.6 (80.5–82.7)		18.4 (17.3–19.5)		< 0.001
30–59		76.3 (75.5–77.0)		23.7 (23.0-24.5)		
60 or older		65.7 (64.5–66.9)		34.3 (33.1–35.5)		
Sex						
Male		77.8 (77.0-78.6)		22.2 (21.4–23.0)		< 0.001
Female		72.1 (71.3–72.9)		27.9 (27.1–28.7)		
Mother tongue						
Non-native		73.4 (72.7–74.1)		26.6 (25.9–27.3)		< 0.001
Native		78.4 (77.2–79.6)		21.6 (20.4–22.8)		
Educational level						
Up to primary		76.1 (75.1–77.0)		23.9 (23.0-24.9)		< 0.001
Secondary		75.9 (74.9–77.0)		24.1 (23.0-25.1)		
Higher		71.2 (70.1–72.3)		28.8 (27.7–29.9)		
Chronic disease or discomfort						
No		83.7 (82.9–84.5)		16.3 (15.5–17.1)		< 0.001
Yes		68.4 (67.6–69.2)		31.6 (30.8–32.4)		
Presence of any permanent limitation or difficulty						
No		75.3 (74.7–75.9)		24.7 (24.1–25.3)		< 0.001
Yes		65.8 (63.6–68.0)		34.2 (32.0-36.4)		
Geographic region						
Coast		69.2 (68.2–70.2)		30.8 (29.8–31.8)		< 0.001
Highlands		81.2 (80.3–82.1)		18.8 (17.9–19.7)		
Jungle		80.1 (78.9–81.2)		19.9 (18.8–21.1)		
Area of residence						
Rural		85.0 (84.2–85.7)		15.0 (14.3–15.8)		< 0.001
Urban		71.6 (70.8–72.3)		28.4 (27.7–29.2)		
Health insurance						
No		85.8 (84.8–86.7)		14.2 (13.3–15.2)		< 0.001
Seguro Integral de Salud		77.2 (76.4–78.0)		22.8 (22.0-23.6)		
Others**		61.0 (59.8–62.2)		39.0 (37.8–40.2)		
Wealth quintile						
I (Poorest)		87.1 (86.2–88.0)		12.9 (12.0-13.8)		< 0.001
II		78.9 (77.7–80.0)		21.1 (20.0-22.3)		
III		74.0 (72.8–75.2)		26.0 (24.8–27.2)		
IV		70.0 (68.6–71.3)		30.0 (28.7–31.4)		
V (Richest)		60.5 (58.8–62.1)		39.5 (37.9–41.2)		

CI: confidence Interval

*P value calculated using the chi-square test with Rao-Scott correction

**Others category was made up of EsSalud, private health insurance, health provider entity, Armed Forces and the Police

Estimates include the expansion factor and sample design characteristics of the ENAHO 2019

**Fig. 2:**
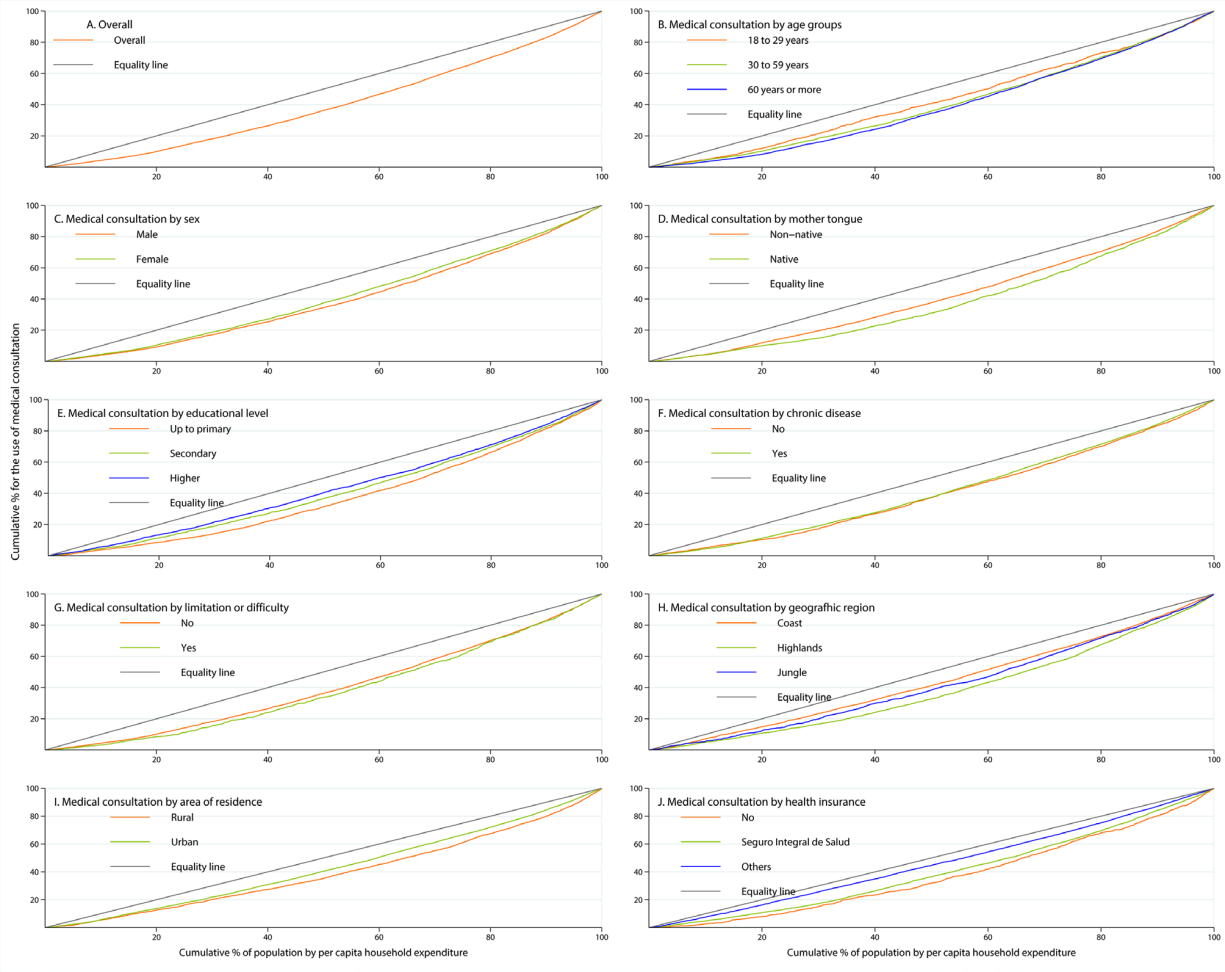
Concentration curves for the use of medical consultation in Peru, ENAHO 2019. *Others category was made up of EsSalud, private health insurance, health provider entity, Armed Forces and the Police

The CCs are shown in Fig. 2. According to the curves, a pro-rich trend was identified for all population subgroups and in general, for all participants. In panel B, the CCs report that inequality for medical consultation among individuals aged 60 years and older is higher compared to younger individuals. In addition, native speakers and those with primary education, those living in the highlands, and those living in rural areas reported greater inequalities compared to non-native speakers, those with higher educational attainment, those living in areas other than the highlands, and those living in urban settings (see panel D, panel E, panel H, and panel I, respectively).

The concentration indexes are shown in Table 3. Overall, the positive value of ECI (0.199) indicates an inequality in favor of the wealthy in the use of medical consultation. A positive value was also reported in all subgroup analyses. When we reported the ECI by population subgroups, we found no differences in inequality in the use of medical consultation by sex (*p* = 0.323) or language (*p* = 0.064). We found differences in inequality in the use of medical consultation according to age (*p* < 0.001), educational level (*p* < 0.001), chronic disease (*p* < 0.001), physical limitations (*p* < 0.001), region of residence (*p* < 0.001), area of residence (*p* < 0.001), and health insurance coverage (*p* = 0.007).

**Table 3:** Concentration index for the use of medical consultation in Peru, ENAHO 2019.

Characteristics	Concentration index	Standard error	*p* value
Overall	0.19937786	0.00641731	< 0.001
Age group (in years)			
18–29	0.10229971	0.01200811	< 0.001
30–59	0.18457323	0.00830868	
60 or older	0.30017989	0.01241237	
Sex			
Male	0.19279535	0.00878072	0.323
Female	0.2046995	0.00825363	
Mother tongue			
Non-native	0.1869384	0.00753137	0.064
Native	0.21440483	0.012779	
Educational level			
Up to primary	0.24694375	0.00967085	< 0.001
Secondary	0.18295935	0.01082359	
Higher	0.17058395	0.01242945	
Chronic disease or discomfort			
No	0.12307242	0.00835029	< 0.001
Yes	0.21884463	0.00865588	
Presence of any permanent limitation or difficulty			
No	0.19277562	0.00652823	< 0.001
Yes	0.31467097	0.02252226	
Geographic region			
Coast	0.14996957	0.01006312	< 0.001
Highlands	0.17383264	0.00834938	
Jungle	0.13146833	0.01440896	
Area of residence			
Rural	0.12322651	0.00764309	< 0.001
Urban	0.16016063	0.00802702	
Health insurance			
No	0.1426812	0.01037717	0.007
Seguro Integral de Salud	0.17825844	0.00896788	
Others*	0.12509796	0.0133331	

*Others category was made up of EsSalud, private health insurance, health provider entity, Armed Forces and the Police

Estimates include the expansion factor and sample design characteristics of the ENAHO 2019

Table 4 shows the decomposition of the ECI according to the covariates included in the study. For the elasticity findings, the value for the female sex was 0.09, which means that a change between male and female increases, by 9%, the probability of widening inequality in the use of medical consultations. In addition, a change from having none to having a chronic disease increases the probability of widening inequality by 23%. On the other hand, a change in the region of residence from coast to highlands reduced the probability of widening inequality by 6%. For the concentration indices, we found that the use of medical consultations for people aged 60 years or older, people with higher education, people with a chronic disease, people living in urban areas and people with other health insurance was concentrated among the richest. In contrast, the use of medical consultations for those who speak their mother tongue and SIS affiliates was concentrated among the poorest. Finally, the main factor of inequalities in the use of medical consultations are having other health insurance, belonging to the richest wealth quintile, having a chronic disease, living in urban environments and residing in the highlands of Peru. Conversely, the main factor in reducing inequality was SIS coverage.

**Table 4:** Decomposition of the concentration index for the use of medical consultation in Peru, ENAHO 2019

Characteristics	Elasticity	Concentraton index	Absolute	%
Age group (in years)				
18–29	Reference	Reference	Reference	
30–59	0.0512	-0.0533	-0.0027	-1.4
60 or older	0.0859	0.0166	0.0014	0.7
Sex				
Male	Reference	Reference	Reference	
Female	0.0922	-0.0012	-0.0001	-0.1
Mother tongue				
Non-native	Reference	Reference	Reference	
Native	0.0046	-0.2769	-0.0013	-0.6
Educational level				
Up to primary	Reference	Reference	Reference	
Secondary	0.0024	-0.0049	0.0000	0.0
Higher	-0.0004	0.423	-0.0002	-0.1
Chronic disease or discomfort				
No	Reference	Reference	Reference	
Yes	0.2394	0.1275	0.0305	15.3
Presence of any permanent limitation or difficulty				
No	Reference	Reference	Reference	
Yes	0.0099	-0.0279	-0.0003	-0.1
Geographic region				
Coast	Reference	Reference	Reference	
Highlands	-0.0686	-0.395	0.0271	13.6
Jungle	-0.0184	-0.0878	0.0016	0.8
Area of residence				
Rural	Reference	Reference	Reference	
Urban	0.1559	0.4842	0.0755	37.9
Health insurance				
No	Reference	Reference	Reference	
Seguro Integral de Salud	0.2472	-0.5296	-0.1309	-65.7
Others*	0.2087	0.4489	0.0937	47.0
Wealth quintile				
I (Poorest)	Reference	Reference	Reference	
II	0.0625	-0.3175	-0.0199	-10.0
III	0.0881	0.0309	0.0027	1.4
IV	0.1069	0.3772	0.0403	20.2
V (Richest)	0.1172	0.5598	0.0656	32.9
Subtotal				91.8
Residual	0.0162815			

*Others category was made up of EsSalud, private health insurance, health provider entity, Armed Forces and the Police

Estimates include the expansion factor and sample design characteristics of the ENAHO 2019

## Discussion

This study estimated inequalities in the utilization of medical consultations in Peru and identified the factors that explain inequalities in utilization. It found that, in 2019 in Peru, 25.4% of people used medical consultations during the last 4 weeks, although their utilization was concentrated among the wealthiest individuals. When decomposing these inequalities, it was found that the greatest contributors were having another type of health insurance (EsSalud, private health insurance, health provider entity, Armed Forces and the Police), residing in an urban area, belonging to the richest wealth quintile, having a chronic disease and residing in the highlands of Peru.

The prevalence of health services utilization reported in this study (25%) is lower than that reported in studies conducted in Italy (28%) [35], Brazil (95.3%) [36], Jamaica (62.9%), Mexico (64.7%), Panama (58.1%) [37], Paraguay (66.5%) [38] and Argentina (40.6%) [39]. These differences between the figures of the countries and our findings could be attributed to the time horizon for measuring the utilization of medical consultations, since most studies are based on the utilization of medical consultations in the last 12 weeks. In addition, a study conducted in Peru reported a higher proportion of utilization of outpatient medical consultation in 2011 (34.8%) than reported in our study, however, this figure was obtained from people who had health insurance (SIS, EsSalud, Armed Forces or private), while people who did not have health insurance only had a medical consultation in 19.1%, which could expose that the possession of health insurance would be a determining factor in the use of health services [40]. Additionally, our finding agreed with that reported in the study conducted by Ypanaqué-Luyo, where women, people affiliated with health insurance, people at the extremes of life, residents of the coast and with chronic diseases had a higher use of a medical consultation [40]. On the other hand, we should note that, in low- and middle-income countries, people are exposed to barriers that could limit the use of health services and not allow adequate health coverage [41]. These factors are related to lack of education, cultural barriers (social stigma, lack of decision-making power, language or traditional medicine preference), poor perception of health services, and low income, which would worsen health status and disease burden in individuals, especially for diseases that require continuous health care [41]. Existing strategies should be strengthened to improve health systems and ensure the universal health coverage required to reduce inequalities in people’s health and the economic impact on countries.

According to the analysis of inequalities, the utilization of a medical consultation was concentrated among the wealthiest individuals. These results are similar to those reported in studies conducted in Chile [14], Uruguay [13], Kenya [42], Italy [35], China [11] and Paraguay [38], where the use of health services was concentrated among people with greater economic resources. The findings are aligned with the hypothesis that wealth has a directly proportional relationship with access to timely and quality health services for individuals, resulting in better long-term health outcomes [43, 44]. On the other hand, the poorest people present barriers that prevent access to adequate health care, due to difficulty in meeting health expenses and distrust in services [45]. This problem has been reported in Peru, where people with lower economic resources had 28 percentage points more problems of access to health services than people with greater resources [46]. This gap has been addressed by the health system through Emergency Decree 017-2019 that seeks to expand health coverage by narrowing the gap for people who do not have health insurance [47]; however, our data will not reflect the impact of this measure on the use of health services due to its early incorporation.

In relation to the decomposition analyses, the major contributors to this inequality were having another type of health insurance (EsSalud, private health insurance, health provider, Armed Forces and the Police), residing in an urban area, belonging to the richest wealth quintile, having a chronic disease, and residing in the highlands of Peru. According to previous studies, having health insurance would increase the probability of seeking outpatient medical care and reduce out-of-pocket spending on health, which would allow adequate access to medical consultation [48]. In addition, people residing in rural areas have a lower probability of having medical care due to long distances between the health facility and the place of residence and low economic income due to their type of occupation (mainly agriculture and livestock) [49]. Likewise, the use of health services in people with chronic diseases is based on the need for long-term and complex management to control their diseases, and on the number of diseases they suffer from, being that people with multimorbidity have a higher use of health services [50]. Finally, it highlights a finding contrary to what has been reported in the literature, where people residing in the highlands contribute to this pro-rich inequality. This finding could reflect an improvement in the healthcare system in this region, which is observed with the steady increase in the number of physicians per 1000 inhabitants between 2009 and 2016 [51].

The findings of this study would have an impact on existing public policies in the Peruvian territory. Although the utilization of medical consultations increased between 2015 and 2019, this increase does not reflect improvements in inequalities in utilization. Under the Universal Health Insurance Framework established in 2009, our results provide an overview of the inequality gap that exists between the poor and the rich in order to redouble efforts to insure populations living in poverty and extreme poverty. Furthermore, this inequality gap will allow government institutions to base their strategies on the main contributors to this socioeconomic inequality in order to achieve greater health coverage and reduce the health and economic impact of diseases on the population. Finally, the Peruvian health system should carry out routine preventive-promotional programs and increase the capacity of health facilities in rural areas to decrease the burden of disease and morbidity and mortality in these underserved populations.

The present study used a survey with nationwide representativeness, which allows us to extrapolate our findings to the entire Peruvian territory. However, it has some limitations. First, the questions asked to the respondents are based on specific events of the past, which could lead to a recall bias. Second, other variables that could be useful for understanding these inequalities were not included due to their unavailability in the database. These variables are related to self-perception of health status, cultural perception of health systems, waiting time to care or community-level variables (such as distance to a health facility). While beliefs and conceptions of health are undoubtedly influential, they often involve deeply ingrained cultural and subjective elements that are challenging to capture accurately through surveys or data collection. Moreover, these factors can be highly context-specific and vary significantly among different population groups, making it difficult to generalize findings. Secondly, including such qualitative and subjective elements would introduce a level of complexity that might complicate the statistical analysis and interpretation of results, potentially obscuring the core factors that we aimed to investigate. Lastly, our theoretical model that underpin our analysis is based on individual determinants that influences the access and use of healthcare services [52]. The model has no power to assess community-level variables. Analyzing those variables requires different research objectives and another theoretical model that supports our analyses. In the Peruvian context, future research endeavors should delve into these nuances to gain a more comprehensive understanding of healthcare utilization inequalities. Finally, although we have analyzed recent data (2019), the changes produced by the COVID-19 pandemic could generate a change in our findings because this health crisis generated a restructuring of healthcare in Peru, especially in populations with a continuous requirement for outpatient care, so it would be necessary to conduct studies that evaluate the change in the time of health care during this health crisis.

## Conclusions

It was found that 25 out of every 100 Peruvians who had a disease accessed a medical consultation during the four weeks prior to ENAHO 2019. In addition, medical consultation was concentrated in people with greater economic resources, where the main contributors were related to socioeconomic and geographic determinants. Despite the fact that, government institutions have sought to increase health coverage in the Peruvian population, there is still a socioeconomic gap between the poor and the rich. Therefore, the search for more equitable access to health services must consider the main contributors to this inequality in order to lessen the negative impact of inadequate disease control in the population.

## Data Availability

The data supporting the findings of this study are publicly available on the website of the National Institute of Statistics and Informatics (INEI) (https://proyectos.inei.gob.pe/microdatos/). The databases can be obtained by accessing the “Survey Query” tab, selecting the “ENAHO Metodología ACTUALIZADA” option under “Survey”. Then, in “(Choose a Survey)” select “Living Conditions and Poverty - ENAHO”, for the year 2019, annual period.

## Abbreviations

LAC: Latin America and the Caribbean
PAHO: Pan American Health Organization
ENAHO: National Household Survey on Living Conditions and Poverty
SIS: Seguro Integral de Salud
EsSalud: Peruvian Social Security
INEI: National Institute of Statistics and Informatics
HIV: Human Immunodeficiency Virus
CC: Concentration curves
ECI: Erreygers concentration index
CI: Confidence interval
